# AI-guided design and optimization of a novel KIM-1-targeted peptide for bFGF delivery in acute kidney injury repair

**DOI:** 10.1093/rb/rbag050

**Published:** 2026-03-09

**Authors:** Kaiyan Su, Zhuo Liu, Pengrui Fu, Runxue Zhou, Hao Xia, Weihong Nie, Shuwei Sun, Chunying Shi, Wei Chen

**Affiliations:** Department of Human Anatomy, Histology and Embryology, School of Basic Medicine, Qingdao University, Qingdao 266071, China; Department of Human Anatomy, Histology and Embryology, School of Basic Medicine, Qingdao University, Qingdao 266071, China; Department of Human Anatomy, Histology and Embryology, School of Basic Medicine, Qingdao University, Qingdao 266071, China; Department of Human Anatomy, Histology and Embryology, School of Basic Medicine, Qingdao University, Qingdao 266071, China; Department of Urology of Jiangbei Campus, The First Affiliated Hospital of Army Medical University (The 958th Hospital of Chinese People’s Liberation Army), Chongqing 400020, China; Department of Medicine, Qingdao University, Qingdao, Shandong Province 266071, China; Department of Human Anatomy, Histology and Embryology, School of Basic Medicine, Qingdao University, Qingdao 266071, China; Department of Human Anatomy, Histology and Embryology, School of Basic Medicine, Qingdao University, Qingdao 266071, China; Department of Urology of Jiangbei Campus, The First Affiliated Hospital of Army Medical University (The 958th Hospital of Chinese People’s Liberation Army), Chongqing 400020, China

**Keywords:** acute kidney injury, basic fibroblast growth factor, targeted drug delivery, artificial intelligence

## Abstract

Acute kidney injury (AKI) remains a major clinical challenge due to its high incidence and mortality rates. Growth factor therapy has emerged as a promising strategy in AKI treatment, but it is limited by low targeting efficiency. Kidney injury molecule (KIM-1) is up-regulated after AKI and serves as a potential target for growth factor delivery systems. In this study, *de novo* designed WEV peptide targeting KIM-1 was screened to systematically optimize the affinity and specificity using a structure-based computational ‘anchor extension’ strategy integrated with deep learning approaches. The WEV peptide was computationally predicted and experimentally validated to have superior specificity and binding affinity with KIM-1 *in vitro* and *in vivo*. Then, a KIM-1-targeted recombinant WEV-bFGF protein was constructed to direct bFGF specifically to the ischemic kidney, which highly expressed KIM-1.This targeted delivery of WEV-bFGF could protect ischemic kidney, decrease cell apoptosis and inflammatory response, alleviate fibrosis and improve renal function. This process was revealed to activate tissue regeneration-related genes, while inhibiting genes related to apoptosis and inflammation, as determined by transcriptome analysis. Therefore, this optimized WEV peptide held a potential for a KIM-1 targeted growth factor delivery system and provided a new therapeutic strategy for AKI treatment.

## Graphical Abstract

**Graphical Abstract:**
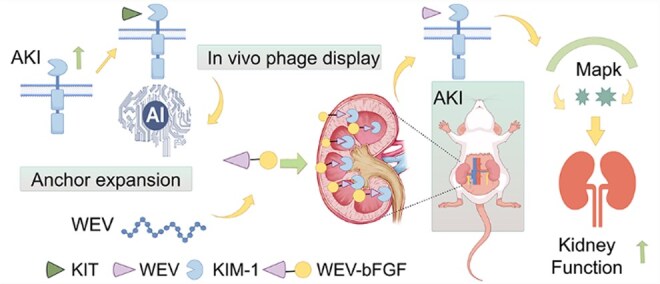

## Introduction

Acute kidney injury (AKI) refers to acute renal dysfunction resulting from a marked reduction or interruption in renal blood perfusion. Its pathophysiological features are primarily characterized by a substantial decline in glomerular filtration function, decreased urine output and the accumulation of nitrogenous metabolites [1]. Therefore, the early restoration of effective renal perfusion and the implementation of renal protective strategies are crucial in clinical practice.

In recent years, regenerative therapies based on growth factors have emerged as promising approaches for the treatment of AKI [2]. Basic fibroblast growth factor (bFGF) [3], a potent mitogenic agent, plays a critical role in inhibiting apoptosis of renal tubular epithelial cells and enhancing renal function in ischaemia/reperfusion (I/R) treatments. By binding to fibroblast growth factor receptor (FGFR), bFGF activates downstream signaling pathways, including PI3K/Akt and MAPK/ERK [4], thereby promoting the survival and proliferation of renal tubular cells. Additionally, bFGF facilitates the regeneration of capillaries surrounding renal tubules, improving renal microcirculation and alleviating hypoxia-induced damage [5]. Currently, conventional bFGF administration for I/R treatment is typically delivered through intraperitoneal, intravenous or intrarenal injections, with the limitations of low targeting efficiency, rapid diffusion and increased potential risks [6–8].

Kidney injury molecule (KIM-1) is a well-established biomarker for early AKI. It is expressed at low levels in normal renal tissue but is significantly up-regulated following renal injury. This characteristic also makes it a potent target for delivering growth factors or drugs to injured kidneys. In a previous study, a specific targeted peptide KIT peptide (CNWMINKEC) was screened out through *in viv*o phage display technology [3]. And we fused the KIT peptide with bFGF to construct recombinant KIT-bFGF proteins. This KIT-bFGF could specifically recognize KIM-1 *in vitro* and *in vivo,* and guide bFGF to the ischemic kidney, thus promoting the functional recovery of AKI [3]. It provided evidence for the effectiveness and feasibility of a KIM-1-targeted, kidney-specific delivery system for bFGF.

In order to increase the specificity and binding affinity, computer-aided drug design (CADD) has focused on optimizing targeted peptide interactions with their binding protein [9–11]. In this study, *de novo* designed peptides targeting KIM-1 were screened to systematically optimize the affinity and specificity. And a structure-based computational ‘anchor extension’ strategy was integrated with deep learning for peptide design. This approach utilized the three-dimensional structure of KIM-1 and the known KIT peptide to extend a *de novo* peptide chain around a non-canonical amino acid residue serving as an anchor [12]. Using this computational design and optimization strategies, a novel peptide—WEV (WEVFGNKEY) was identified to have superior specificity and binding affinity with KIM-1. Then the targeting abilities of this peptide were verified through *in vitro* hypoxia/reoxygenation models of HK-2 cells and *in vivo* AKI model of rats, each of which exhibited upregulated KIM-1 expression in injured kidney cells and tissues correspondingly. Finally, the WEV peptide was fused with bFGF by gene engineering to construct WEV-bFGF targeting delivery system. And the biological and targeting capacity of WEV-bFGF were evaluated *in vitro* and *in vivo*. After establishing a rat renal ischemia model, the therapeutic effects and molecular mechanism of WEV-bFGF were further investigated.

## Materials and methods

### Spatial locus prediction

Firstly, the three-dimensional structures of the target protein KIM-1 and the known binding peptide KIT were predicted using the AlphaFold 2.3 module on the WeMol platform, and the corresponding PDB files were generated. Subsequently, molecular docking between KIM-1 and KIT was performed using HDOCK to obtain the complex structure (KIM-1–KIT complex), from which the key binding region NKE within KIT was identified as the structural anchor for subsequent design. Based on this anchor, saturation mutagenesis was performed at the six non-anchor sites across the KIT sequence to construct a preliminary library containing 114 single-point mutants. The change in binding affinity of each mutant to KIM-1 was evaluated by calculating the mutation score of binding, and the two amino acid residues with the most favorable binding score at each position were selected. Using a Python-based exhaustive combinatorial algorithm, these optimal residues were incorporated into the original sequence framework, resulting in 64 candidate peptide sequences. The three-dimensional structure of each candidate peptide was predicted using AlphaFold 2.3, and molecular docking with KIM-1 was conducted via HDOCK to assess binding affinity. The conformational stability of each docked complex was evaluated by calculating the root mean square deviation (RMSD) to ensure structural reliability. Based on docking scores and RMSD values, a comprehensive ranking was performed to identify candidate peptides with improved binding compared to the wild-type KIT. Finally, the top five peptide sequences with the highest comprehensive scores and better conformational stability were selected, and their binding modes with KIM-1 were further validated through molecular docking.

### Peptide synthesis

The peptides Negative control (NC, amino acid sequence: NSSSVDK [13]), KIT (Amino acid sequence: CNWMINKEC) and WEV (Amino acid sequence: WEVFGNKEY) were designed by the Laboratory of Human Anatomy at Qingdao University and chemically synthesized by Sangon Biotech (Shanghai, China). In addition, for fluorescence distribution detection, the peptides were modified with 5-Carboxytetramethylrhodamine (5-TAMRA) and for quantitative determination, they were modified with Biotin. All peptide samples were purified to ≥98% purity using standard reversed-phase high-performance liquid chromatography (RP-HPLC).

### *In vitro* hypoxia/reoxygenation model of HK-2 cells

Human renal tubular epithelial cells (HK-2) were purchased from ATCC (American Type Culture Collection, USA) and routinely cultured at 37°C and 5% CO_2_ in DMEM high glucose medium containing 10% fetal bovine serum and 1% penicillin-streptomycin. Subsequently, HK-2 cells were seeded into 48-well plates at a cell density of 3–5 × 10^4^ cells per well. After the cells adhered, the original culture medium was discarded and high-glucose complete medium mixed with 5-TAMRA-labeled peptides (50 ng/mL) was added separately, and cultured under hypoxic conditions (1% oxygen, 5% carbon dioxide, 94% nitrogen) for 12 h, followed by reoxygenation culture for 3 h. The fluorescent observation was performed to evaluate the targeting ability of different peptides in hypoxic HK-2 cells. And fluorescence images acquired under consistent parameters were quantified using ImageJ for further statistical analysis.

### Construction of a rat renal ischemia-reperfusion injury model

All animal experiments were approved by the Animal Ethics Committee of Qingdao University (Approval No QDU-AEC-2023365) and complied with the ‘Guidelines for the Care and Use of Laboratory Animals’. Healthy adult female SD rats (220 ± 20 g, Jinan Peng Yue Laboratory Animal Breeding Co., Ltd) were fasted for 12 h (with free drinking water) before surgery. Under anesthesia by intraperitoneal injection of pentobarbital sodium (40 mg/kg), a midline abdominal incision (2–3 cm) was made to expose the kidneys. The unilateral renal artery was clamped for 45 min using a non-invasive microvascular clip, with the abdominal cavity covered with 37°C normal saline gauze. After reperfusion for 60 min, 200 μg of 5-TAMRA-labeled peptides were injected via the tail vein. At 6 and 24 h post-injection, peptide distribution was assessed using the small animal *in vivo* fluorescence imaging system (IVIS Spectrum, Perkin Elmer, USA). Rats were then sacrificed and major organs (heart, liver, lungs and kidneys) were collected for *ex vivo* imaging. Ischemic kidneys were frozen at −80°C for sectioning, and fluorescence distribution was observed under a fluorescence microscope (IX83, Olympus, Tokyo, Japan) and analyzed with ImageJ.

For evaluating the renal protective effects of recombinant proteins, the AKI model rats were randomly divided into four groups: WEV-bFGF (15 μg, 150 μL), KIT-bFGF (15 μg, 150 μL), native bFGF (15 μg, 150 μL) and PBS (150 μL). Recombinant proteins or PBS were injected after reperfusion. To assess targeting, kidneys and serum were collected at 6 and 24 h post-injection. bFGF levels were measured using the human bFGF ELISA kit (EK0441, Boster, China), and renal function was analyzed with a standard Creatinine assay kit (C011-2-1, Nanjing Jiancheng, China).

### Construction of WEV-bFGF recombinant protein

The WEV sequence was fused to the N-terminus of bFGF cDNA through chemical synthesis by Sangon Biotech (Shanghai) Co., Ltd. The recombinant plasmid pET-28a (+)-WEV-Linker-bFGF (Novagen, USA) was transformed into *Escherichia coli* BL21(DE3) and cultured at 37°C, followed by induction with 1 mM isopropyl-β-D-thiogalactoside for 5 h. Recombinant bFGF, KIT-bFGF and WEV-bFGF were purified using an ÄKTA nickel column protein purification system (GE Healthcare, UK). Purity and yield were assessed by 15% SDS-PAGE. The spatial structures of WEV-bFGF and natural bFGF were predicted based on the bFGF sequence template from the NCBI database using the WeMol database and analyzed with Pymol software.

The biological activity of WEV-bFGF was evaluated using human skin fibroblasts (HSFs). Cells were seeded in 96-well plates at 5 × 10³–1 × 10^4^ cells per well. After adherence, the medium was replaced with low-serum (2% FBS) DMEM containing bFGF, KIT-bFGF or WEV-bFGF (100, 50, 25, 12.5 and 6.25 ng/mL), with six replicates per concentration. After 24 h, proliferation was assessed using CCK-8 reagent by measuring absorbance at 450 nm. The protective effects of the recombinant proteins were further evaluated in an *in vitro* hypoxia/reoxygenation model using HK-2 cells. Cells were seeded in 48-well plates at 0.6–1 × 10^4^ cells/well. After adhesion, they were treated with bFGF, KIT-bFGF or WEV-bFGF (12.5–50 ng/mL) and subjected to hypoxia (1% O_2_, 5% CO_2_, 94% N_2_) for 12 h followed by 3 h reoxygenation. Cell viability was assessed using CCK-8 kits (GK10001, GLPBIO, USA). Additionally, recombinant WEV-bFGF, KIT-bFGF and native bFGF were crosslinked with Dylight-800 (#46621, Thermo Fisher Scientific, USA) for targeting evaluation in hypoxic HK-2 cells and ischemic kidney tissues as described in ‘In vitro hypoxia/reoxygenation model of HK-2 cells’ and ‘Construction of a rat renal ischemia-reperfusion injury model’.

### ELISA

After the establishment of the renal ischemia-reperfusion injury model, tail vein injection was used to inject WEV-bFGF (15 μg, 150 μL), KIT-bFGF (15 μg, 150 μL) or natural bFGF (15 μg, 150 μL) into rats in sequence. At the two time points of 6 and 24 h after injection, kidney tissues and serum samples from the ischemic side of rats were collected separately. Subsequently, in accordance with the instructions of the Human bFGF ELISA Kit (EK0441, Boster, China), the protein extracted from kidney tissue and the content of bFGF in serum were determined.

### Renal function assessment after renal ischemia-reperfusion

Renal function was evaluated using serum creatinine (Scr). After establishing renal ischemia-reperfusion injury, the animals were divided into four groups: WEV-bFGF (15 μg, 150 μL), KIT-bFGF (15 μg, 150 μL), natural bFGF (15 μg, 150 μL) and PBS (150 μL). Then the corresponding protein was injected through the tail vein. Rat sera were collected at 24 h and 2 weeks after administration, and the creatinine (Cr) detection kit (C011-2-1, Nanjing Jiancheng, China) was used to evaluate renal function.

### Western blot

To extract total protein, 0.05 g of kidney tissue was taken and lysed in 0.5 mL of RIPA lysis buffer containing 5 μL of PMSF. After the determination of protein concentration, it was separated by SDS-PAGE electrophoresis and transferred to PVDF membrane. The membrane was incubated with blocking solution with 5% skimmed milk at room temperature for 1–2 h. The primary antibody was incubated using rabbit anti-bFGF polyclonal antibody (1:1000, BS6432, Bioworld, China) overnight at 4°C. Tris-buffered saline with Tween 20 (TBST) was thoroughly washed and horseradish peroxidase-labeled goat anti-rabbit secondary antibody (1:10 000, HA1001, HUABIO, China) was incubated at room temperature for 1 h. Ultimately, detection and image acquisition were carried out on the chemiluminescence imager (Tanon-5200) using hypersensitive chemiluminescence reagents (SQ201, YaEnzyme, China).

### H&E and Masson

After the animals were sacrificed, the kidneys were harvested and paraffin sections were prepared. For H&E staining, sections were dewaxed, rehydrated and stained with Harris hematoxylin and eosin. After dehydration and mounting, images of the corticomedullary junction were captured under a Nikon Eclipse E100 microscope (40× objective) using the NIS-Elements system. Renal injury was scored on a 0–5 scale based on tubular damage extent across 10 non-overlapping fields (40× objective) under double-blind conditions. The specific scoring criteria were as follows: 0, normal kidney tissue; 1, minimal damage (≤10% of tubules affected); 2, mild damage (11–25)%; 3, moderate damage (26–45)%; 4, severe damage (46–75)%; 5, very severe damage (≥76%). The assessment was based on characteristic histopathological features including tubular epithelial necrosis, tubular dilation, loss of brush border and cast formation. For Masson’s trichrome staining, dewaxed sections were stained with Weigert’s hematoxylin, Lichun red, phosphomolybdic acid and aniline blue to visualize collagen deposition. After dehydration and mounting, images were captured under a Nikon Eclipse Ni microscope (20× objective). Collagen volume fraction in the renal interstitium was quantified from 10 corticomedullary fields per sample using Image-Pro Plus 6.0.

### Immunofluorescence staining

After dewaxing, rehydration and antigen retrieval, paraffin sections were blocked with 20% fetal bovine serum (SH30406, Solarbio, China) for 30 min to minimize non-specific binding. The sections were then incubated with a primary antibody mixture consisting of rabbit anti-KIM-1 (1:300, NBP1-76701, Novus, USA), mouse anti-FGF2 (1:200, sc-74412, Santa Cruz Biotechnology, USA), rabbit anti-cleaved Caspase-3 antibody (1:700, #9664, Cell Signaling Technology, USA), rabbit anti-CD68 (1:500, HA722285, HUABIO, China) and rabbit anti-CD31 (1:200, HA720372, HUABIO, China) at 4°C overnight. Following three washes with PBS (5 min each), the sections were re-blocked with 10% serum for 15 min. Subsequently, they were incubated in the dark with secondary antibodies: anti-rabbit IgG-Alexa Fluor 594 (1:500, ab150064, Abcam, UK) and anti-mouse IgG-Alexa Fluor 488 (1:500, ab150105, Abcam, UK) at room temperature for 1 h. Nuclei were stained with DAPI (1 μg/mL), and the slides were mounted using anti-fade mounting medium (ProLong Diamond, P36961, Invitrogen, USA). The image was performed using a confocal microscope in sequential scanning mode to avoid cross-talk. Co-localization analysis was conducted with the ImageJ plugin Coloc2, and Pearson’s correlation coefficient (Rr > 0.5 indicated significant co-localization) was calculated. Quantitative data were obtained from at least three independent experiments.

### TUNEL staining

After dewaxing and rehydration, paraffin sections were treated with proteinase K (20 μg/mL, 37°C, 20 min) and processed using the TUNEL apoptosis detection kit (Alexa Fluor 488, 702410ES03, Yeasen, China) per manufacturer’s instructions. Sections were incubated with TdT enzyme reaction buffer (37°C, 60 min, dark), washed with PBS and mounted with DAPI-containing antifade reagent (P36961, ProLong Diamond, Invitrogen). Imaging was performed on a laser confocal microscope (Leica TCS SP8) using sequential scanning (488 nm for TUNEL, 405 nm for DAPI). Apoptotic rates were calculated from five non-overlapping cortical fields per section as (TUNEL^+^ nuclei/total DAPI nuclei) × 100% using ImageJ.

### Transcriptome sequencing and biological analysis

In order to further explore the molecular mechanism of WEV-bFGF in AKI, transcriptomic analysis was carried out. Specifically, samples were collected 24 h after the recombinant protein injection, and eukaryotic transcriptome sequencing was performed by Sangon Biotech. Subsequently, the differential gene expression map was constructed using bioinformatics methods. Furthermore, the expressions of key genes including *Bak*, *Mapk1*, *Il6r*, *Mapk6*, *Nfkb*ie, *Fgf2*, *Bcl212* and *Casp8* were validated by qPCR (Supplementary Table S1). Briefly, RNA was extracted from three biologically independent replicates using the TransZol Up Plus Kit (ER501-01-V2, Transgen). Following this, genomic DNA was eliminated, and cDNA was synthesized immediately using the EasyScript One-Step gDNA Removal and cDNA Synthesis SuperMix (AE311-02, Transgen). Finally, qPCR reactions were prepared with the PerfectStart Green qPCR SuperMix (AQ601-01-V2, Transgen) and primers from Sangon Biotech in a 20-µL volume and were run on a Bio-Rad CFX96 Touch instrument in accordance with the manufacturer’s guidelines.

### Statistical analysis

Quantitative data are expressed as mean ± standard deviation. Statistical evaluation was performed using GraphPad Prism (version 8.0) and SPSS 26.0. One-way ANOVA was used to analyze the differences in renal function indicators (serum creatinine), histopathological scores (H&E, Masson) and apoptosis markers (TUNEL^+^ cells, cleaved caspase-3^+^ cells) among the groups. The comparisons between paired groups (determination of the biological activities of WEV-bFGF, KIT-bFGF and natural bFGF and the survival rate of HK-2 cells) were conducted using the bilateral unpaired *t*-test; *P *< 0.05. It was defined as statistical significance.

## Results

### Spatial site docking and *in vitro* targeting ability detection

**Figure 1:**
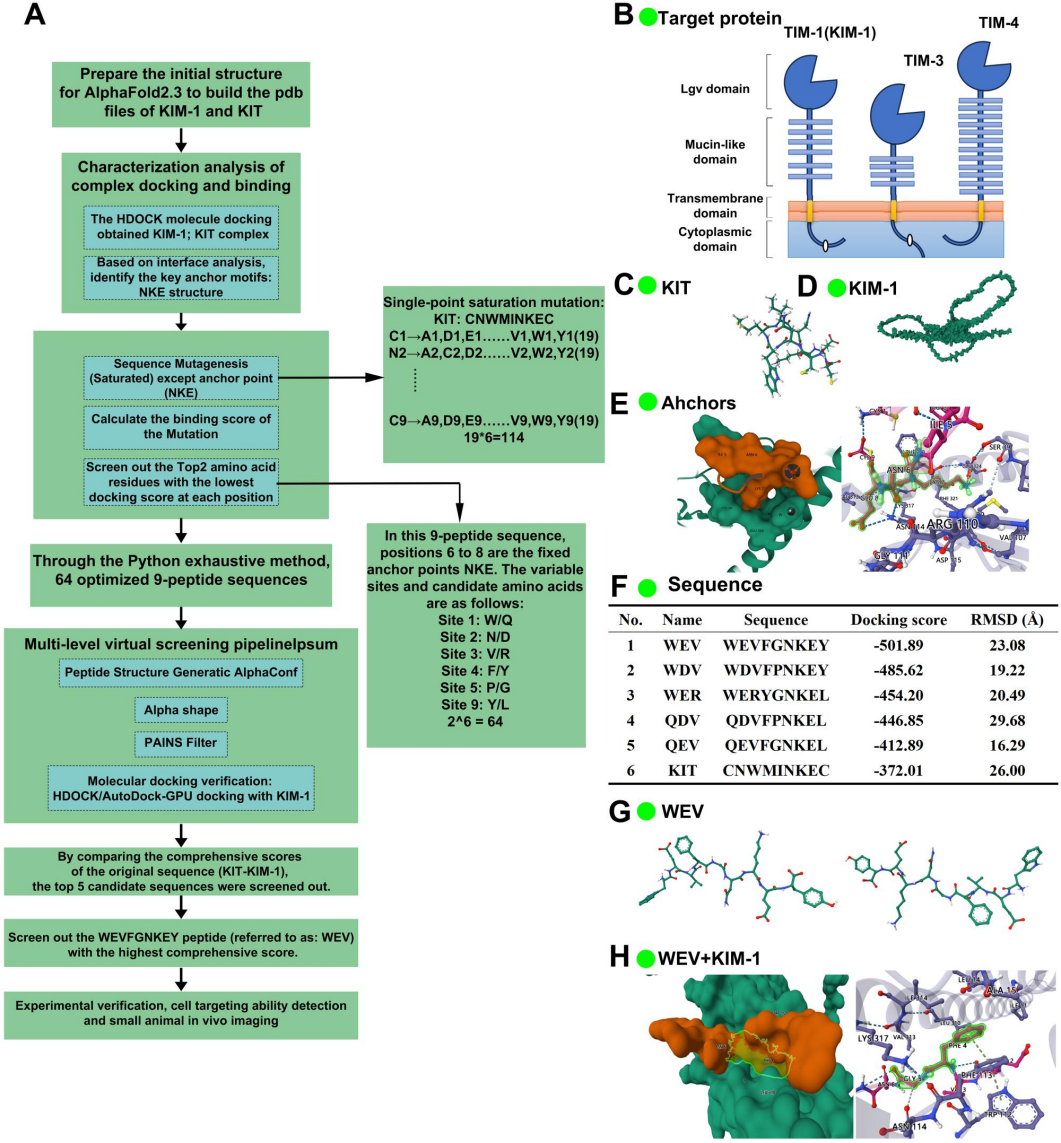
The computational design and optimization of KIM-1 targeted peptides. (**A**) Overall workflow of the computational peptide design, screening and validation process employed in this study; (**B**) Structural representation of the KIM-1 domain (surface model); (**C**) Molecular structure of the KIT; (**D**) Molecular model of the KIM-1 protein; (**E**) Analysis of the binding interface between KIM-1 and KIT, highlighting the conserved ‘NKE’ motif as the key anchor point for interaction; (**F**) Optimized peptide sequences with their corresponding binding score and root-mean-square deviation (RMSD, Å) values; (**G**) Molecular model of the optimized peptide WEV; (**H**) Interaction analysis of the WEV-KIM-1 binding interface.

This study aimed to design high-affinity peptides targeting the IgV domain of KIM-1. Based on the interaction between KIM-1 and the known KIT peptide, we established a computational peptide design pipeline (Figure 1A). KIM-1 was a transmembrane protein with an extracellular region, transmembrane region and intracellular region (Figure 1B). The three-dimensional structures of KIM-1 and the KIT peptide were predicted using AlphaFold2 (Figure 1C and D). Molecular docking with HDOCK generated a complex structure, showing the KIT peptide directly bound to the IgV domain of KIM-1, a key region for recognizing extracellular ligands. Structural analysis revealed the NKE motif as a crucial binding anchor (Figure 1E). The binding score of KIT to KIM-1 was −372.01. We then designed novel KIM-1 targeting peptides through amino acid saturation mutagenesis. Single-site saturation mutagenesis was performed on the KIT sequence (excluding the anchor site). The two amino acids with the lowest binding score at each site were selected to construct a 64-peptide library using the Python exhaustive method (general formula: X_1_X_2_X_3_X_4_X_5_NKEX_9_). All peptides were subjected to structure prediction and docking with KIM-1 using AlphaFold2.3 and HDOCK. Finally, five candidates with higher binding scores and better conformational stability than KIT were screened (Figure 1F).

Based on binding scores and conformational stability, the WEV peptide (WEVFGNKEY) with a score of −501.89 and RMSD of 23.08 Å was selected. Molecular docking indicated a superior spatial binding pattern with KIM-1 (Figure 1H). Specifically, Phe4 of WEV formed a π-π stacking with Phe113 of KIM-1, and Gly5 formed a hydrogen bond with Asn114 of KIM-1. In contrast, the KIT peptide formed a hydrogen bond network via its NKE anchor: Asn6 (OD1) with Asn114 (ND2), Lys7 (NZ) with Glu32 (OE2) and Glu8 (OE2) with Asn114 (ND2) of KIM-1. These findings indicate that the WEV peptide exhibits superior binding by forming a more extensive interaction network with KIM-1.

### The evaluation of *in vitro* and *in vivo* targeting ability of the WEV peptide

**Figure 2:**
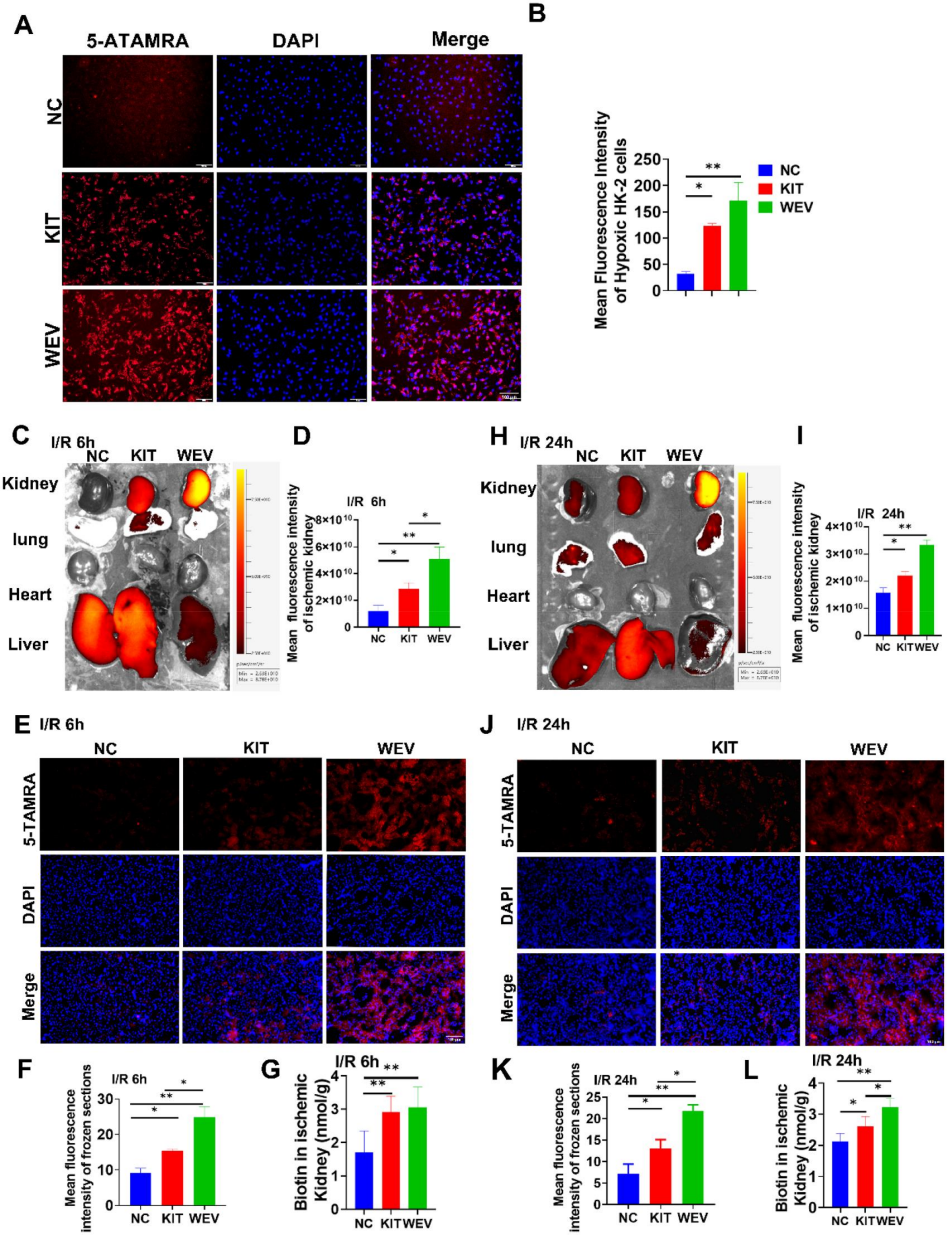
The *in vivo* and *in vitro* targeting capabilities of the targeted peptide WEV. (**A**) Fluorescence distribution of 5-TAMRA-labeled peptides in hypoxic HK-2 cells; (**B**) Statistics on the average fluorescence intensity of 5-TAMRA-labeled peptides in hypoxia-reperfusion HK-2 cells; (**C**) At 6 h after administration, the *in vivo* fluorescence distribution of 5-TAMRA-labeled targeted peptides in major organs of AKI-injured rats by the small animal imaging system; (**D**) Statistical analysis of fluorescence intensity of 5-TAMRA-labeled peptides in ischemic kidneys at 6 h after administration; (**E**) The distribution of 5-TAMRA-labeled peptides in ischemic renal tissue by frozen section observation at 6 h after AKI injury, Scale bar = 50 μm; (**F**) Statistical analysis of fluorescence intensity in kidney frozen sections at 6 h after administration; (**G**) The content of Biotin-labeled peptides in ischemic kidney at 6 h after injection; (**H**) At 6 h after administration, the *in vivo* fluorescence distribution of 5-TAMRA-labeled targeted peptides in major organs of AKI-injured rats by the small animal imaging system; (**I**) Statistical analysis of fluorescence intensity of 5-TAMRA-labeled peptides in ischemic kidneys at 24 h after administration; (**J**) The distribution of 5-TAMRA-labeled peptides in ischemic renal tissue by frozen section observation at 24 h after AKI injury, Scale bar = 50 μm; (**K**) Statistical analysis of fluorescence intensity in kidney frozen sections at 24 h after administration; (**L**) The content of Biotin-labeled peptides in ischemic kidney at 24 h after injection. All quantitative data are presented as mean ± SD, *P < 0.05, **P < 0.01, *N* = 6.

In the previous study, it was revealed that KIM-1 was upregulated after AKI injury in both HK-2 cells and the kidney [14]. And *in vitro* hypoxia/reoxygenation model was established using HK-2 cells, which were then incubated with various 5-TAMRA-labeled peptides. The targeting ability of the WEV peptide with KIM-1 was assessed based on the fluorescence distribution in the hypoxic HK-2 cells. As shown in Figure 2A and B, the fluorescence density of WEV peptide and KIT peptide was upregulated compared to the NC peptide with statistical difference. And then the *in vivo* AKI model of rats was established, various 5-TAMRA-labeled peptides were injected through the tail vein, and the fluorescence intensity was assessed using *in vivo* imaging at 6 and 24 h post-injection. The results of fluorescence distribution at 6 h after injection, the fluorescence intensity of the WEV peptide was the strongest in ischemic renal tissue compared with those in KIT peptide and the NC peptide, which had a significant statistical difference. And there was also notably higher fluorescence intensity in KIT peptide group than NC peptide (Figure 2C and D). These results were further confirmed by fluorescence analysis of kidney frozen sections after *in vivo* live imaging observation (Figure 2E and F). Moreover, the fluorescence distribution was also detected in other major organs, with intense signals in the liver, moderate levels in the lungs, and negligible distribution in the heart. And the fluorescence signals in the liver were also statistically evaluated, which the NC group had higher fluorescence intensity than WEV peptide and KIT peptide (Supplementary Figure S1A). In addition, similar results were obtained in both *in vivo* live imaging and frozen kidney sections at 24 h after injection (Figure 2H and I; Supplementary Figure S1B). Therefore, these results indicated that the WEV peptide specifically bound to KIM-1, and exhibited superior targeting capability than KIT peptide, as evidenced in both hypoxic HK-2 cells *in vitro* and in ischemic kidney tissues *in vivo* with high KIM-1 expression. Finally, quantitative assessment was performed by Biotin-labeled peptides. Consistent with the results of *in vivo* fluorescence distribution, the detectable biotin content in the ischemic kidney was generally elevated in both the WEV and KIT peptide groups compared to the NC peptide group, with a significant difference at 6 h post-injection (Figure 2G). At 24 h post-injection, the biotin level in the WEV peptide group remained considerably higher than both the KIT and NC peptide groups. Additionally, the KIT peptide group also showed statistically higher biotin content than the NC peptide group (Figure 2L). These results revealed the optimized WEV peptide had superior binding affinity with KIM-1 as validated in both *in vitro* hypoxia/reoxygenation model of HK-2 cells and *in vivo* rat model of AKI, suggesting its potential for targeted drug delivery in the treatment of AKI.

### The construction of KIM-1 targeted bFGF delivery system

**Figure 3:**
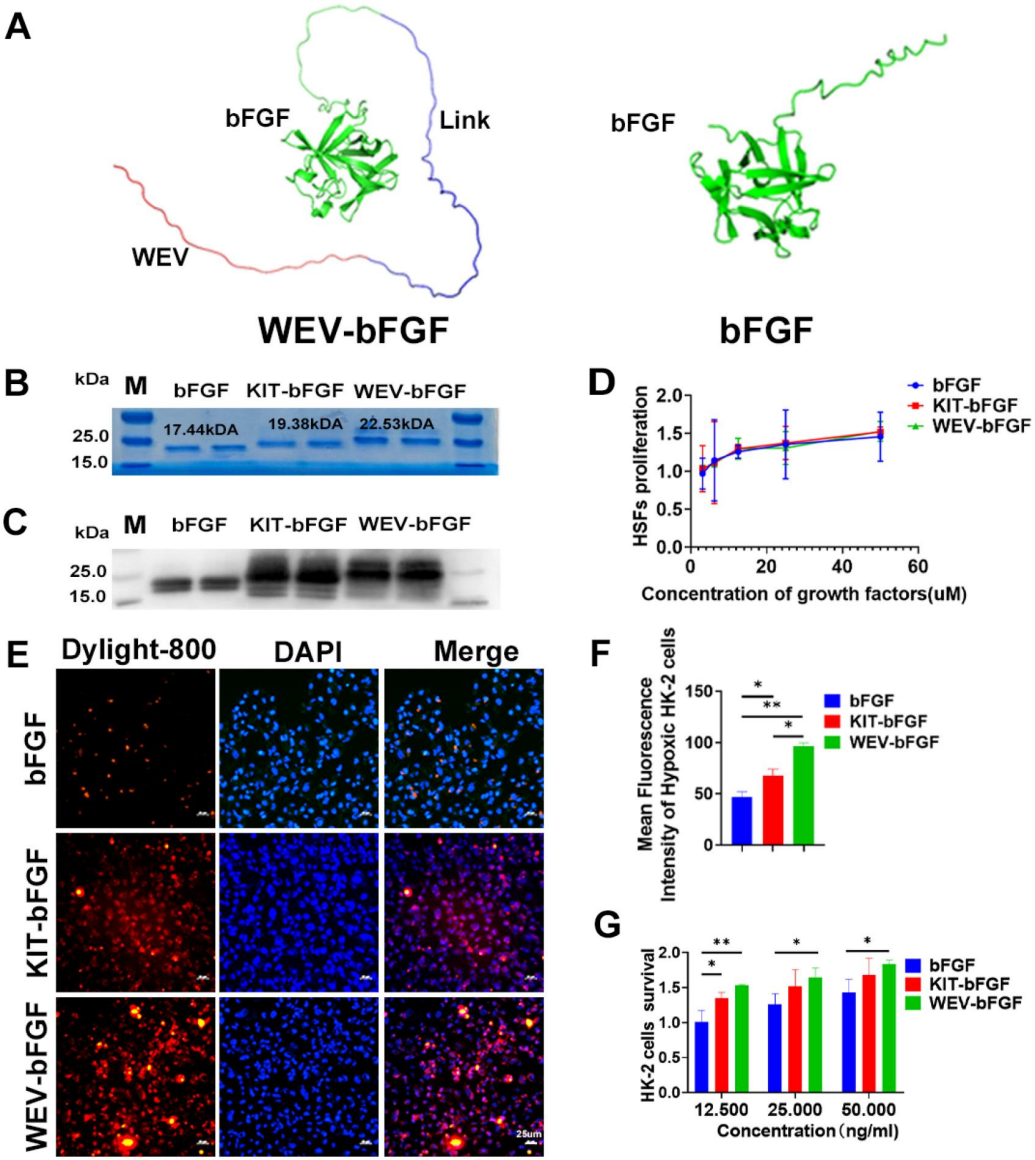
Construction and bioactivity evaluation of WEV-bFGF. (**A**) Spatial structure prediction of WEV-bFGF recombinant protein and natural bFGF; (**B**) SDS-PAGE of bFGF, KIT-bFGF and WEV-bFGF; (**C**) Western blot of native bFGF, KIT-bFGF and WEV-bFGF; (**D**) The biological activities of native bFGF, KIT-bFGF and WEV-bFGF by CCK-8 assay; (**E**) The fluorescence distribution of Delight 800 labeled recombinant proteins in hypoxic HK-2 cells; (**F**) Statistical analysis of fluorescence intensity of Dylight 800 in hypoxic HK-2 cells; Scale bar = 25 μm; (**G**) The protective effects of recombinant proteins on HK-2 cells after hypoxic injury by the CCK8 assay. All quantitative data are presented as mean ± SD, *P < 0.05, **P < 0.01.

As the targeted peptide WEV could effectively enriched in hypoxic renal cell and tissue with high expressed KIM-1, the recombinant WEV-bFGF was constructed by fusing the WEV peptide and bFGF with linker. Firstly, the homologous modeling analysis based on the WeMol cloud platform indicated that the introduction of WEV peptide did not change the three-dimensional conformation of the core active domain of bFGF (Figure 3A). After purification by affinity chromatography, SDS-PAGE and western blot were used to examine both native bFGF (17.44 kDa) and WEV-bFGF (22.53 kDa) (Figure 3B and C). Biological activity verification showed that after treating with different concentrations of WEV-bFGF, KIT-bFGF and native bFGF for 24 h, the proliferation rates of HSFs were similar (Figure 3D), indicating that the fusion of KIT or WEV peptides did not affect the biological activities of bFGF. Both *in vitro* and *in vivo* experiments had shown that the WEV peptide had a good targeting ability in hypoxic HK-2 cells and ischemic renal tissues. Then the recombinant proteins were modified with Dylight-800 respectively and whether the recombinant WEV-bFGF protein could also target and bind with hypoxic HK-2 cells was evaluated. As shown in Figure 3E and F, the WEV-bFGF exhibited higher fluorescence intensity and more specific localization compared with KIT-bFGF and native bFGF, which had significant difference. Additionally, KIT-bFGF also showed statistically higher fluorescence intensity than the bFGF group. Thus, the protective effects of WEV-bFGF were also detected via hypoxia/reoxygenation model of HK-2 cells. The results showed that at a concentration of 12.5 ng/mL, the cell survival rate in the WEV-bFGF-treated group was higher than those in both the KIT-bFGF and native bFGF groups; and at high concentrations of 25 and 50 ng/mL, WEV-bFGF could protect hypoxic HK-2 cells than native bFGF group (Figure 3G), indicating WEV-bFGF might be a promising candidate for targeted therapy of AKI.

### The assessment of WEV-bFGF accumulation in ischemic kidneys of rats after intravenous injection

**Figure 4:**
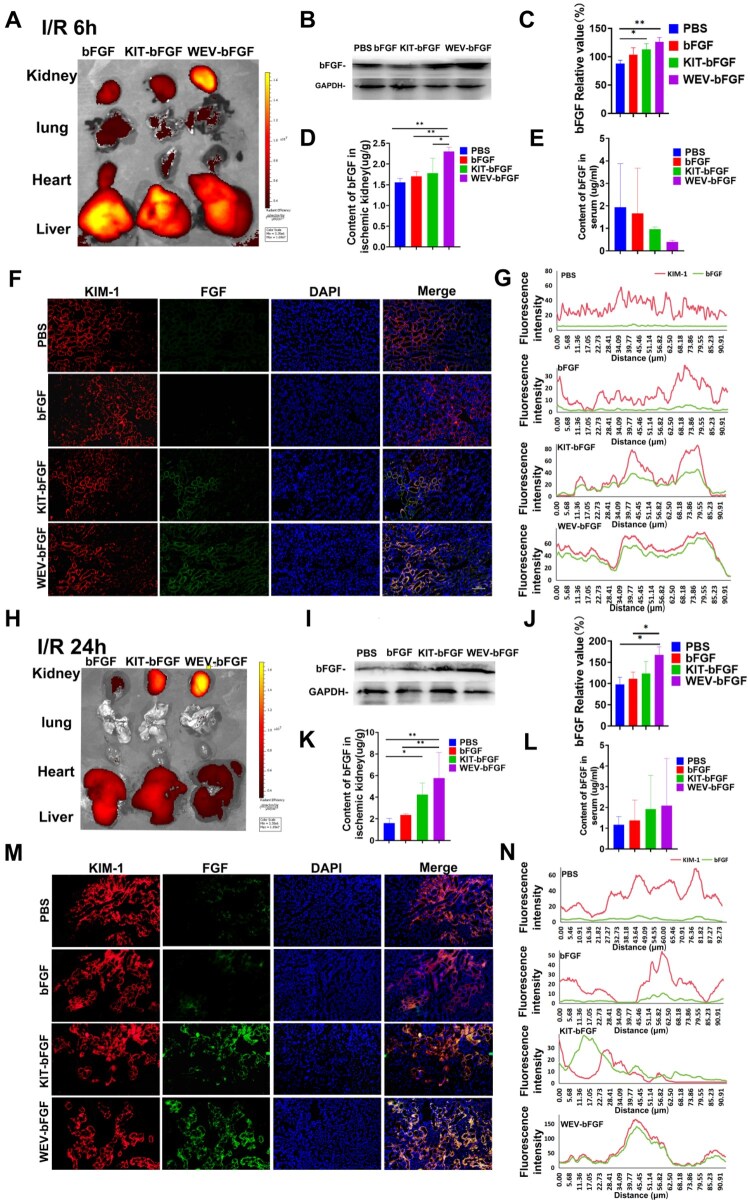
The evaluation of targeting capacity of WEV-bFGF in ischemic kidney *in vivo*. (**A**) At 6 h after administration, the *in vivo* fluorescence distribution of Dylight800-labeled recombinant proteins in major organs of AKI-injured rats by the small animal imaging system; (**B**) Western blot showing bFGF protein expression in ischemic renal tissue 6 h after administration; (**C**) Quantitative analysis of relative bFGF protein expression at 6 h; (**D**) The content of bFGF in ischemic kidneys by ELISA assay at 6 h after administration; (**E**) The content of bFGF in serum at 6 h after administration; (**F**) Immunofluorescence colocalization of bFGF and KIM-1 in the frozen sections at 6 h after administration, Scale bar = 50 μm; (**G**) Colocalization analysis of PBS, bFGF, KIT-bFGF and WEV-bFGF groups at 6 h after administration; (**H**) At 24 h after administration, the *in vivo* fluorescence distribution of Dylight800-labeled recombinant proteins in major organs of AKI-injured rats by the small animal imaging system; (**I**) Western blot showing bFGF protein expression in ischemic renal tissue 24 h after administration; (**J**) Quantitative analysis of relative bFGF protein expression at 24 h; (**K**) The content of bFGF in ischemic kidneys by ELISA assay at 24 h after administration; (**L**) The content of bFGF in serum at 24 h after administration; (**M**) Immunofluorescence staining showing colocalization of bFGF and KIM-1 at 24 h after administration, scale bar = 50 μm; (**N**) Colocalization analysis of PBS, bFGF, KIT-bFGF and WEV-bFGF groups at 24 h after administration. All quantitative data are presented as mean ± SD, *P < 0.05, **P < 0.01, *N* = 8.

Given the confirmed *in vitro* and *in vivo* targeting ability of WEV peptides, their potential to enrich bFGF in the ischemic kidney was further evaluated. The recombinant native bFGF, KIT-bFGF and WEV-bFGF were cross-linked with Dylight 800. After establishing the renal ischemia model, the Dylight 800-labeled recombinant proteins were intravenously injected and the fluorescence distribution was observed at 6 and 24 h post-injection. Through the *in vivo* imaging system, the WEV-bFGF group showed a significant difference in the average fluorescence intensity of the kidneys compared with the bFGF group at the 6 h post-injection, whereas there was no significant difference between KIT-bFGF group and bFGF group (Figure 4A; Supplementary Figure S2A). In other major organs, the fluorescence signals were mainly detected in liver, lungs and heart, with more content in bFGF group compared with WEV-bFGF group and KIT-bFGF group (Supplementary Figure S2B). After gross observation, the ischemic kidneys were taken out for further fluorescence distribution observation in frozen section. As shown in Supplementary Figure S2C and D, the fluorescence intensity of the WEV-bFGF group and KIT-bFGF was upregulated compared with bFGF group. These results were confirmed by western blot as shown in Figure 4B. Then the contents of bFGF in ischemic kidney and serum were quantified by ELISA. At 6 h after injection, the content of bFGF in ischemic kidney of WEV-bFGF group (2.30 ± 0.11 μg/g) was considerably higher than that in KIT-bFGF group (1.78 ± 0.36 μg/g), bFGF group (1.70 ± 0.12 μg/g) and PBS group (1.56 ± 0.09 μg/g), and there was no statistical difference among these four groups in serum (Figure 4D and E). Additionally, due to KIT and WEV peptides specifically bound to KIM-1, the immunofluorescence co-localization of bFGF and KIM-1 was performed in ischemic kidney to evaluate their interaction (Figure 4F). After AKI, the KIM-1 was notably upregulated in each group but with no statistical difference. However, the expression of bFGF in the KIT-bFGF and WEV-bFGF groups showed a significant difference from the bFGF and PBS groups (Supplementary Figure S2E). Furthermore, the statistical analysis of co-localization showed that WEV-bFGF had the most obvious co-localization of KIM-1 and bFGF than the other three groups, and KIT-bFGF also had better co-localization of KIM-1 and bFGF compared with bFGF and PBS (Figure 4G).

At 24 h after injection, the *in vivo* imaging results showed that there were most obviously immunofluorescence signals detected in WEV-bFGF group compared to KIT-bFGF group and bFGF group (Figure 4H); meanwhile, the immunofluorescence signals could be detected in liver, which had a stronger signal in bFGF group compared to WEV-bFGF group and KIT-bFGF group, but it was undetectable in lungs and heart (Figure 4H; Supplementary Figure S2G). Furthermore, the immunofluorescence distribution observation in ischemic kidney sections showed at 24 h post-injection, the average fluorescence intensity of WEV-bFGF group was notably higher than both KIT-bFGF group and bFGF group, and there was also statistically stronger fluorescence in KIT-bFGF group and bFGF group (Supplementary Figure S2J). And quantitative ELISA results showed the content of bFGF in ischemic kidney of WEV-bFGF group (5.76 ± 2.39 μg/g) was considerably more than that of both bFGF group (2.35 ± 0.11 μg/g) and PBS group (1.60 ± 0.43 μg/g); and the content of bFGF in KIT-bFGF group (4.25 ± 1.09 μg/g) was also considerably higher than PBS group; in serum, there was also no statistical difference among these four groups (Figure 4K and L). These results were further confirmed by western blot and the immunofluorescence co-localization of bFGF and KIM-1 (Figure 4I, J, M and N). Overall, these results indicated that WEV-bFGF could specifically target KIM-1 and effectively retained bFGF in ischemic kidneys after acute injury.

To further clarify the *in vivo* dynamic characteristics of WEV-bFGF, we conducted a systematic assessment of its pharmacokinetic behavior. In the AKI rat model, we quantitatively determined the concentrations of bFGF delivered by WEV-bFGF or KIT-bFGF in serum and kidney tissues at different time points after tail injection (15, 30 min, 1, 3, 6, 12, 24, 48 h) (Supplementary Figure S3A and B). The results showed that the clearance kinetics of bFGF from the two recombinant proteins in the blood circulation was similar, and there was no statistically significant difference in the calculated serum half-life (Supplementary Figure S3C and D).

Given that the recombinant protein we designed mainly targets the KIM-1 that was highly expressed after injury, its distribution in the normal physiological state was expected to be different. Therefore, we compared the metabolic conditions of bFGF delivered by WEV-bFGF and KIT-bFGF in healthy rats (Supplementary Figure S3E and F). ELISA results showed that there was no significant difference in the bFGF concentrations in the serum of healthy rats at 6 and 24 h after injection; more importantly, neither of them showed obvious specific accumulation of bFGF in normal kidney tissues. This result further supports the targeting mechanism that the accumulation of bFGF via WEV-bFGF depends on the high expression of KIM-1 induced by injury.

### The evaluation of renal function and morphology following intravenous WEV-bFGF injection

**Figure 5:**
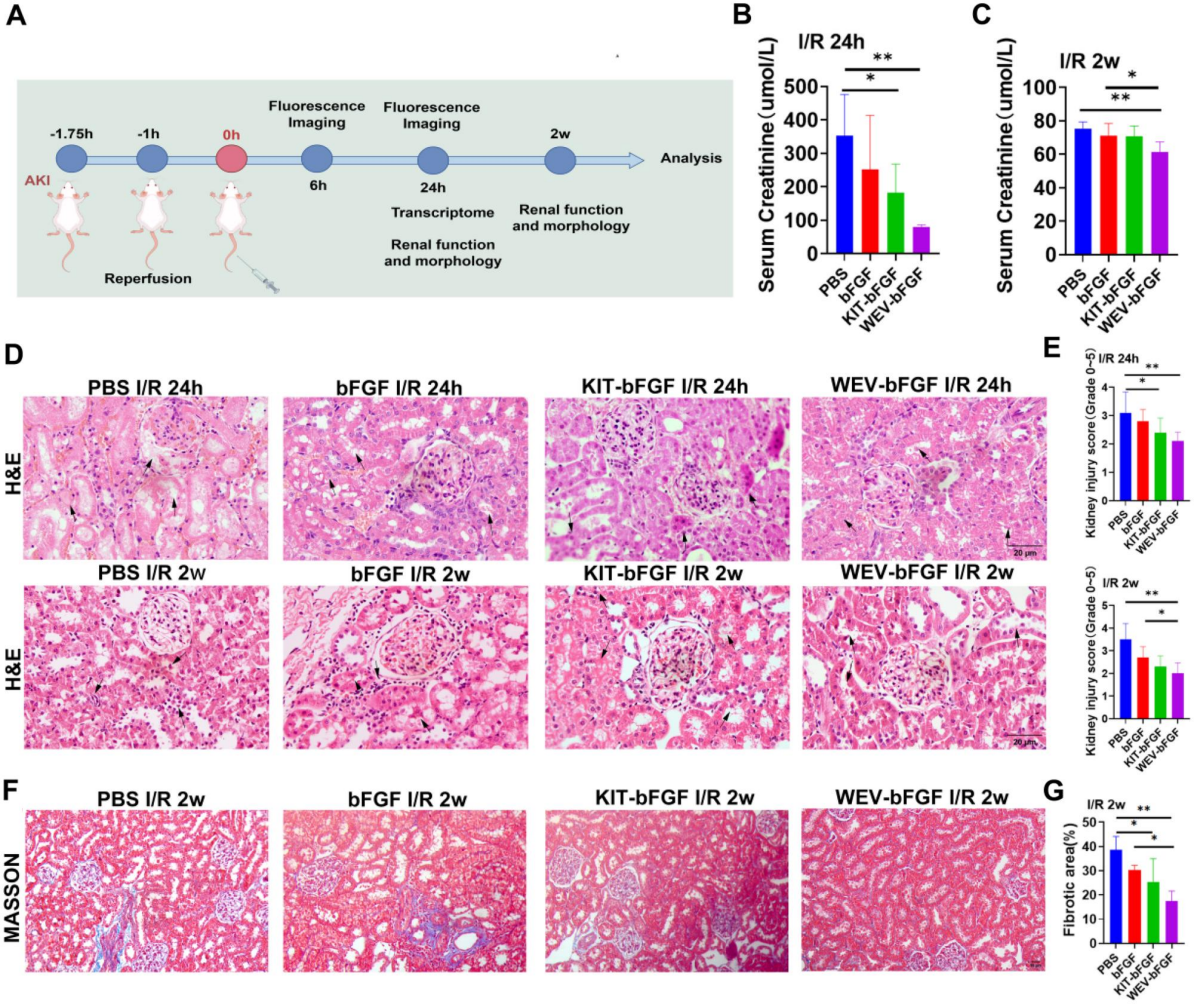
Functional tests and morphological analysis of the kidneys. (**A**) Experimental design of WEV-bFGF for the treatment of I/R; (**B**) Statistical analysis of serum Scr content for renal function evaluation at 24 h after renal I/R injury in rats; (**C**) Statistical analysis of serum Scr content for renal function evaluation at 24 h after renal I/R injury in rats; (**D**) H&E staining for kidney histopathological evaluation after I/R injury. Arrows indicated the sites of renal tubular injury. Scale bar = 20 μm; (**E**) Statistical analysis of renal tubular injury score; (**F**) MASSON staining for renal tissue fibrosis evaluation after I/R injury. Scale bar = 50 μm; (**G**) Statistical analysis of renal tissue fibrosis. All quantitative data are presented as mean ± SD, *P < 0.05, **P < 0.01, *N* = 8.

Due to the preferable binding affinity with KIM-1, WEV-bFGF could effectively accumulate in hypoxic HK-2 cells and ischemic kidney tissues. Therefore, the protective effects of WEV-bFGF on rats with AKI were further explored. The experimental design was described in Figure 5A. Firstly, the kidney function was detected through the serum creatinine test. As shown in Figure 5B and C, the serum creatinine level in the WEV-bFGF group (79.43 ± 7.19 μmol/L) and KIT-bFGF group (207.99 ± 88.06 μmol/L) was pronouncedly lower than that in that of PBS group (352.5 ± 123.49 μmol/L) at 24 h post-injection; and at 2 weeks after injection, the serum creatinine level in the WEV-bFGF group (63.37 ± 6.83 μmol/L) was pronouncedly lower than that of both bFGF group (68.3 ± 12.6 μmol/L) and PBS group (71.79 ± 6.21 μmol/L). Therefore, these results indicated that the targeted delivery of WEV-bFGF could improve the functional recovery of AKI.

Subsequently, the morphological injury caused by AKI was further evaluated through H&E staining (Figure 5D and E). And the PBS group exhibited typical characteristics of ischemia-reperfusion injury, including: extensive necrosis of renal tubular epithelial cells, obvious tubular formation, significant renal tubular dilation and severe interstitial congestion. In contrast, the renal tubular structure in the WEV-bFGF group and KIT-bFGF was relatively well preserved, with minimal loss of tubular architecture and a significant reduction in interstitial congestion. The quantitative analysis of the renal tubular injury score confirmed the above results (Figure 5E). At 24 h after injection, the injury scores of the WEV-bFGF group (2.10 ± 0.32) were notably lower than those of the bFGF group (2.80 ± 0.42) and the PBS group (3.10 ± 0.73); at 2 weeks after injection, the injury scores of the WEV-bFGF group (2.00 ± 0.47) were still statistically lower than those of the bFGF group (2.70 ± 0.48) and the PBS group (3.50 ± 0.71). It was reported AKI could directly induce cell apoptosis and inflammatory responses, leading to the release of inflammatory mediators such as tumor necrosis factor α (TNF-α), interleukin (IL-1β) and ROS. These mediators further promoted the activation of myofibroblasts, increased the deposition of extracellular matrix and ultimately contributed to progressive kidney fibrosis [15, 16]. And the intervention of the protective bioactive factors such as bFGF in the acute stage could effectively prevent the progression from AKI to chronic kidney disease [17]. Therefore, the kidney fibrosis was detected by Masson’s trichrome staining at 2 weeks after injection (Figure 5F and G). The fibrotic area in the PBS group (38.73 ± 5.41)% was higher than that of in WEV-bFGF group (17.54 ± 4.12)% and t KIM-1 targeted growth factor delivery system he KIT-bFGF group (25.42 ± 9.58)%, but with no significant difference with the bFGF group (30.19 ± 2.02)%. These results indicated that through specifically targeting with KIM-1, the WEV-bFGF could protect kidney against ischemic injury, preserving renal morphology and alleviating the potential fibrosis process.

### The evaluation of renal cell apoptosis following intravenous WEV-bFGF injection

**Figure 6:**
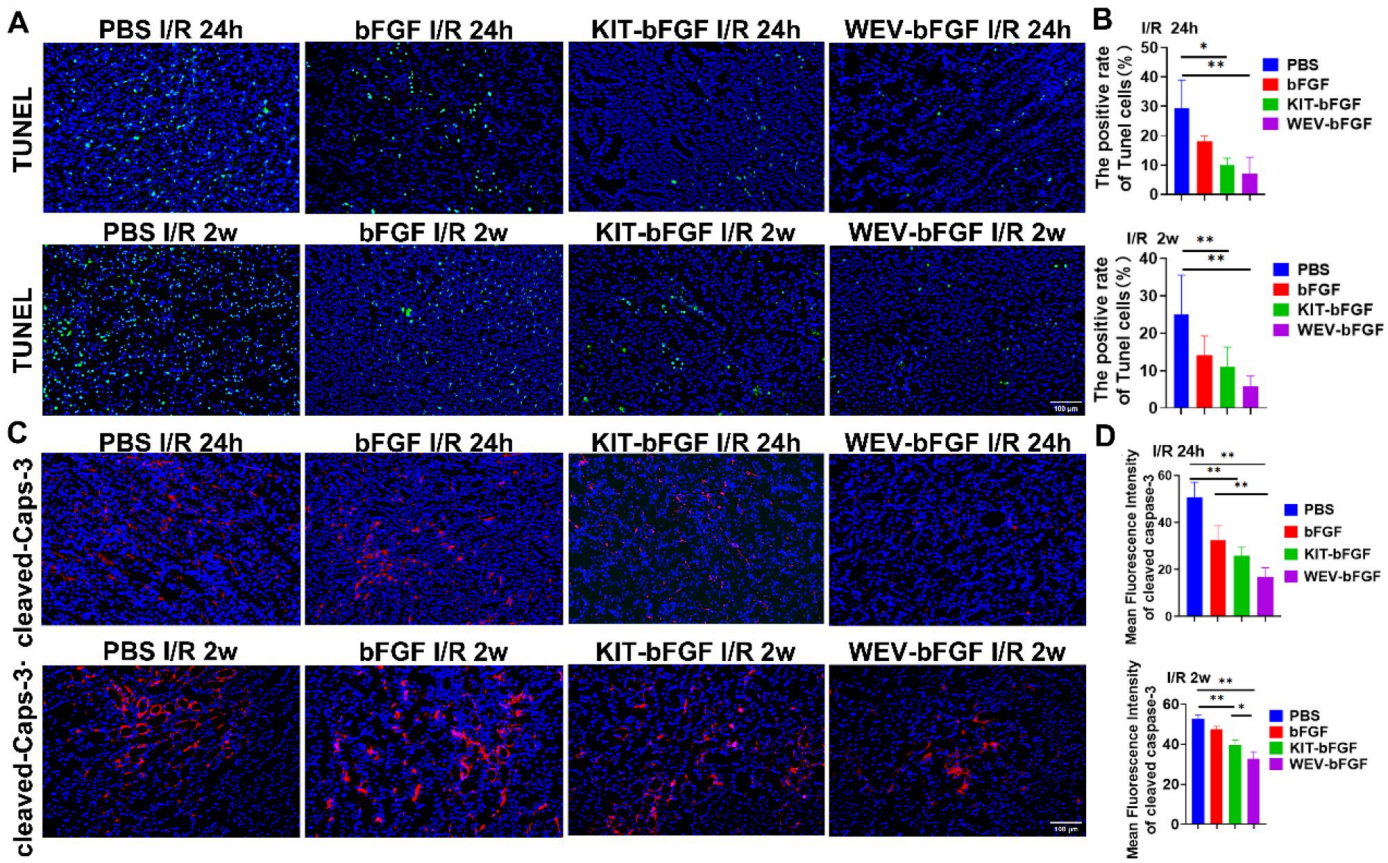
The cell apoptosis assessment. (**A**) TUNEL staining for renal cell apoptosis assessment. Scale bar = 100 μm; (**B**) Statistical analysis of TUNEL-positive cells; (**C**) Immunohistochemical evaluation of cleaved caspase-3 expression. Scale bar = 50 μm; (**D**) Statistical analysis of the mean fluorescence intensity (MFI) of cleaved caspase-3. All quantitative data are presented as mean ± SD, *P < 0.05, **P < 0.01, *N* = 8.

Then, cell apoptosis was further detected by TUNEL staining (Figure 6A and B). The results showed that due to I/R, the cell apoptosis markedly increased in PBS group, and the proportion of TUNEL-positive cells of PBS group (25.08 ± 9.57, 29.29 ± 8.83)% was markedly higher than that of WEV-bFGF group (5.83 ± 2.52, 7.01 ± 5.11)% and KIT-bFGF group (10.97 ± 4.87, 9.85 ± 2.27)%, but with no significant difference with bFGF group (14.12 ± 4.71, 18.05 ± 1.59)% at both 24 h and 2 weeks post-injection respectively.

Since caspase-3 served as a key terminal cleavage enzyme in the apoptotic process, with its activated form (cleaved caspase-3) being activated during early apoptosis [18], we detected cleaved caspase-3 expression at both 24 h and 2 weeks post-injection. The results revealed that the expression of cleaved caspase-3 in WEV-bFGF group and KIT-bFGF group was decreased compared with that of PBS group, which had significant difference as shown in Figure 6C and D. These results indicated that the KIM-1 targeted WEV-bFGF and KIT-bFGF attenuated hypoxia-induced apoptosis by directly suppressing the expression of cleaved caspase-3.

### The detection of inflammatory responses and angiogenesis following intravenous WEV-bFGF injection

Existing literature had shown that renal ischemia could rapidly trigger an inflammatory cascade reaction, thereby exacerbating renal tubular injury and interstitial remodeling [19]. Within several hours of reperfusion, pro-inflammatory cytokines and ROS recruited CD68^+^ macrophages into the kidneys. These infiltrating cells amplified the damage and subsequently drove fibrotic signaling [15, 16]. To evaluate the sustained anti-inflammatory effect of WEV-bFGF, the macrophage infiltration in ischemic kidney was detected by immunofluorescence staining of CD68-positive macrophages. The results showed that 24 h after injection, the percentage of positive cells of CD68 in the WEV-bFGF group was (10.78 ± 2.08)% and that in the KIT-bFGF group was (19.38 ± 1.85)%, which was lower than that in the PBS group (36.10 ± 11.32)%. Two weeks after injection, the inflammatory levels in each group showed a downward trend. Compared with the bFGF group (21.13 ± 3.36)% and the PBS group (29.45 ± 7.78)%, the expression level of CD68 in the WEV-bFGF group (8.71 ± 2.42)% was still lower than that of the former two. The expression level of CD68 in the KIT-bFGF group (17.05 ± 4.37)% was lower than that in the PBS group, and the difference was significant.

In addition, bFGF was a potent angiogenic growth factor [20]. Then the pro-angiogenic effect of WEV-bFGF was also explored by the CD31 immunostaining to detect the capillaries formation. As shown in Figure 6C and D, the number of CD31- positive capillaries in WEV-bFGF group was substantially higher than that in bFGF group and PBS group. At 2 weeks after injection, CD31-positive capillaries revealed a higher fluorescence intensity in the WEV-bFGF group compared to that of bFGF and PBS groups. These data indicated that KIM-1 targeted WEV-bFGF demonstrated sustained advantages in suppressing inflammatory response and promoting angiogenesis, that facilitating the functional recovery of I/R.

### The molecular mechanisms underlying intravenous WEV-bFGF therapy for renal I/R injury repair

Finally, the molecular mechanisms of WEV-bFGF in AKI were explored using transcriptome analysis. As shown in Figure 8A, the analysis of differentially expressed genes (DEG) showed that compared with PBS, 105 genes increased and 132 genes decreased in the WEV-bFGF group. Compared with bFGF, 105 genes were up-regulated and 218 genes were down-regulated in the WEV-bFGF group (Figure 8C). Subsequently, the signaling pathways of tissue repair and regeneration after recombinant proteins administration were analyzed using the KEGG database. Based on the KEGG enrichment analysis, WEV-bFGF group demonstrated significant therapeutic effects compared to of both the PBS group and the bFGF group. In the comparison between WEV-bFGF and PBS, WEV-bFGF significantly upregulated signaling pathways related to tissue synthesis and development, such as Erythroblastic B leukemia viral oncogene homolog (ErbB), Wingless-related integration site (Wnt), Hippo pathway (Hippo) and stem cell pluripotency regulation (Figure 8B). It simultaneously inhibited immune and inflammation-related pathways such as Tumor Necrosis Factor (TNF), Transforming Growth Factor-β (TGF-β) and cytokine-cytokine receptor interactions, thereby promoting the tissue repair process. Compared with the use of bFGF alone, WEV-bFGF significantly upregulated the pathways such as PPAR, estrogen, stem cell pluripotency regulation and sphingolipid metabolism, while inhibiting immune-inflammatory and cell apoptosis signaling pathways such as TNF, interleukin-17 (IL-17), Toll-like receptors and chemokines, further improving the regenerative effect (Figure 8D). These results indicated that WEV-bFGF enhanced the anabolic process to promote the regenerative microenvironment, but also exerted therapeutic potential through synergistic anti-inflammatory effects.

Furthermore, the expression profiles of differentially expressed genes (DEGs) were performed through FPKM (Fragments Per Kilobase of transcript per Million mapped fragments) analysis (Figure 8E). The results demonstrated that compared with other groups, a subset of genes in the WEV-bFGF group including interleukin 17 receptor D (*Il17rd*), caspase 2 (*Casp2*), caspase 8 (*Casp8*), caspase 7 (*Casp7*), nuclear factor of kappa light polypeptide gene enhancer in B-cells inhibitor alpha (*Nfkbia*), BCL2-antagonist/killer (*Bak*) and interleukin 6 receptor (*Il6r*)—were downregulated. Conversely, genes encoding key mitogen-activated protein kinase (MAPK) signaling components, such as mitogen-activated protein kinase 8 (*Mapk8*), mitogen-activated protein kinase 1 (*Mapk1*) and mitogen-activated protein kinase 7 (*Mapk7*), mitogen-activated protein kinase 6 (*Mapk6*) were upregulated. These gene expression changes collectively indicated a suppression of inflammatory and apoptotic processes, coupled with an activation of MAPK-mediated pro-survival and reparative signaling [21, 22], which might contribute to the observed attenuation of renal injury and facilitation of tissue repair. In addition, the key genes involved in transcriptome analysis were verified through qPCR (Figure 8F). The results demonstrated that compared to the PBS group, the WEV-bFGF group exhibited downregulated expression of *IL-6r*, *Nfκbie* and *Casp8* genes while the expression levels of *Mapk1*, *Bcl-212* and *Mapk6* were upregulated. These findings suggest that WEV-bFGF might modulate key pathways involved in tissue regeneration, cell survival and inflammatory response.

## Discussion

Stimulation-responsive drug delivery systems (SRDDSs) could be activated by specific stimuli in the injured microenvironment, allowing for precise drug release at the target site [23, 24]. This targeted approach enhanced therapeutic efficacy while minimizing systemic toxicity. In kidney diseases, these endogenous stimuli usually include pH changes, reactive oxygen species (ROS), enzymes and glucose, which reflected characteristic molecular pathological cues within the diseased microenvironment [25]. In addition, kidney injury biomarkers were another kind of molecular target, which were upregulated after kidney injury and served as an ideal guide for the spatiotemporally controlled drug release. In a previous study, we constructed a KIM-1 targeted delivery system (KIT-bFGF) that specifically delivered bFGF to ischemic renal tissues with high KIM-1 expression, thereby protecting against ischemic injury and promoting renal functional recovery [3].

In recent years, computational design and optimization strategies for targeted peptide screening have made remarkable progress in the fields of drug discovery and tissue engineering [25]. The design strategies for peptides and protein fragments targeting protein binding mainly covered three types of methods: structure-guided design, *de novo* design and machine learning-based design [26, 27]. Structure-guided methods were based on natural binding motifs to enhance binding affinity and specificity through site-directed mutagenesis, cross-linking stabilization (such as helical pinning, cyclization) and scaffold grafting. *De novo* design was divided into sequence-first, structure-first and sequence-structure collaborative optimization strategies, generating novel binding peptides by using physical energy functions (such as Rosetta, EvoDesign) or conformational sampling algorithms (such as GenKIC). Machine learning methods were relied on predictive models (such as CPP, AMP and ACP classifiers) and generative models (such as RNN and CPANN) to optimize peptide sequences and functions through data-driven approaches. The AI-driven computational strategy demonstrated distinct advantages over the traditional *in vivo* phage display methods. It circumvented the necessity for extensive and complex *in vivo* screening procedures, substantially reducing both time and cost. Furthermore, this computational approach integrated target protein structural information and modeled the governing principles of peptide-target interactions, enabling *de novo* generation and precise identification of optimal peptide sequences. Consequently, the resulting peptides exhibited enhanced binding affinity, improved specificity, and superior stability, while simultaneously minimizing off-target potential.

In present study, a theoretically optimal WEV peptide was designed by anchor point expansion based on the three-dimensional structures of KIM-1 and the previously validated KIT peptide. After the key anchor amino acids had been identified, novel KIM-1 targeting peptides were designed and optimized through amino acid saturation mutagenesis and deep learning approaches using the Python exhaustive method. Finally, WEV peptide was screened out to have the superior binding scores and conformational stability compared to the original KIT peptide (Figure 1). The present strategy integrated structure-guided design and machine learning-based approaches, which focused on maximizing the accuracy and stability of the monomeric designed structures, as the binding between KIM-1 and the KIT peptide had been previously confirmed.

Then the effectiveness of WEV peptide was further experimentally assessed. Due to KIM-1 specifically upregulated in hypoxia/reoxygenation model of HK-2 cells and AKI models of rats, the targeting abilities of WEV peptide were firstly verified *in vitro* and *in vivo*. As shown in Figure 2A and B, compared to NC peptide and KIT peptide, 5-TAMRA-labeled WEV peptide showed enrichment on the surface of hypoxic HK-2 cells and ischemic kidney. Then these peptides were modified with Biotin and injected into renal ischemia rats, and compared with the PBS group or the bFGF group, the quantitative biotin content of WEV-bFGF in ischemic kidneys was significant more than that of NC group at 6 h post-injection and also statistically higher than both NC group and KIT group (Figure 2G). These results showed WEV peptide showed superior binding affinity with KIM-1 compared to KIT peptide, suggesting its potential as a preferred targeting ligand for KIM-1-mediated delivery systems in renal ischemia therapy. Subsequently, the WEV peptide was fused with bFGF to construct the recombinant WEV-bFGF protein, which had similar bioactivity with native bFGF (Figure 3A and B). Consistent with the results of WEV peptide, the Dylight 800 modified recombinant WEV-bFGF proteins could specifically target and retain in hypoxic HK-2 cells *in vitro* and ischemic kidney *in vivo* (Figures 3E, 4A and H). And semiquantitative western blot and quantitative ELISA assay provided direct evidence that more bFGF retained in ischemic kidney by targeted with KIM-1 (Figure 4B–E and I–L). In addition, the co-localization fluorescence staining showed WEV-bFGF also had better colocalization with KIM-1 at both 6 and 24 h after recombinant protein injection, achieving the enrichment and long-term retention of bFGF (Figure 4E, F, K and L). To further elucidate the *in vivo* pharmacokinetic profile of WEV-bFGF, the results showed both WEV-bFGF and KIT-bFGF exhibited similar clearance kinetics in systemic circulation, with no statistically significant difference in their serum half-lives (Supplementary Figure S3C and D). Crucially, a controlled study in healthy rats was performed. The results showed no significant difference in serum bFGF concentrations between the two groups at 6 and 24 h post-injection; more importantly, neither group showed obvious specific accumulation of bFGF in normal kidney tissues (Supplementary Figure S3E and F). These findings demonstrated that the renal enrichment of bFGF mediated by WEV-bFGF was dependent on injury-induced KIM-1 overexpression, underscoring the precision and context-dependent nature of its targeting mechanism.

**Figure 7:**
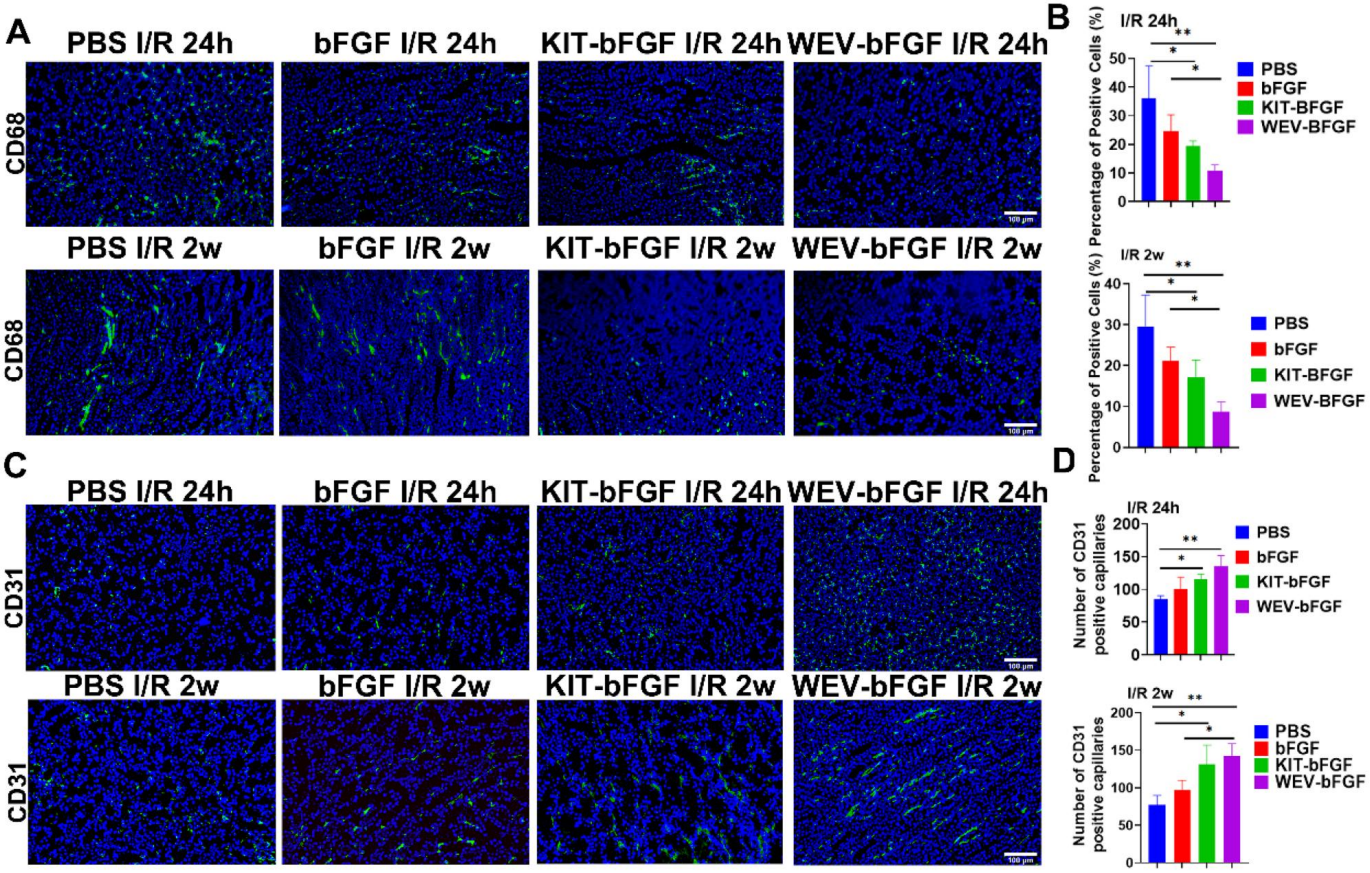
The evaluation of inflammation and angiogenesis. (**A**) CD68 staining for the detection of inflammatory cells infiltration in ischemic kidneys. Scale: 100 μm; (**B**) Statistical analysis of percentage of CD68 positive cells; (**C**) CD31 staining for the detection of capillaries in ischemic kidneys. Scale bar = 100 μm; (**D**) Statistical analysis of the number of CD31-positive capillaries. All quantitative data are presented as mean ± SD, *P < 0.05, **P < 0.01, *N* = 8.

Furthermore, the protective effect of KIM-1-targeted WEV-bFGF delivery system was explored in rats with kidney ischemia. Firstly, the renal function was evaluated by serum creatinine content. The results showed the creatinine content of WEV-bFGF and KIT-bFGF was lower than that of the PBS group at 24 h after injection; and the creatinine content of WEV-bFGF was also markedly lower than that of bFGF and the PBS group at 2 weeks after injection (Figure 5B and C). The pathological staining showed that WEV-bFGF ameliorated morphological damage, attenuated tissue fibrosis, reduced cell apoptosis and suppressed inflammatory responses (Figures 5–7). Therefore, these results indicated that due to its typical interaction with KIM-1 in the injured microenvironment for sustained release of bFGF, the KIM-1-targeted WEV-bFGF could effectively protect kidney against ischemic injury.

**Figure 8:**
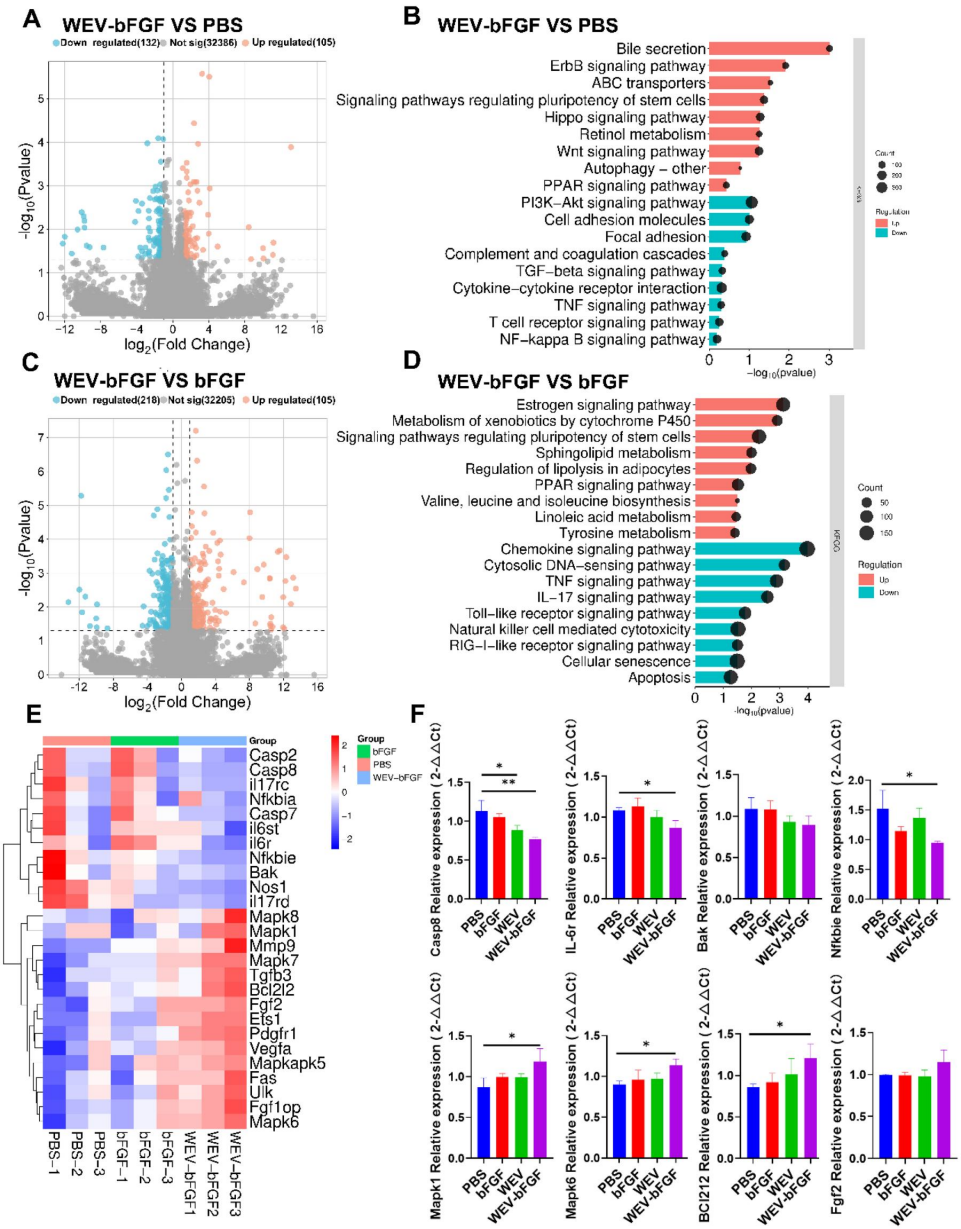
Transcriptome analysis of potential mechanism in WEV-bFGF mediating ischemic renal repair. (**A**) Volcano plots of gene differences between the WEV group and the PBS group; (**B**) KEGG analysis between the WEV group and the PBS group; (**C**) Volcano plots of gene differences between the WEV-BFGF group and the bFGF group; (**D**) KEGG analysis between the WEV-bFGF group and the bFGF group; (**E**) Heat map of differentially expressed genes related to repair and regeneration of AKI; (**F**) Quantitative PCR validation of key gene expression. All quantitative data are presented as mean ± SD, *P < 0.05, **P < 0.01, *N* = 6.

In addition, the potential molecular mechanism of WEV-bFGF in the repair of renal AKI injury was further investigated (Figure 8). Through transcriptome analysis, WEV-bFGF upregulated signaling pathways related to tissue regeneration and development, such as ErbB, Wnt, Hippo and stem cell pluripotency regulatory pathways, while inhibiting immune and inflammation-related pathways, such as TNF, TGF-β and cytokine-cytokine receptor interactions, thereby synergistically promoting the tissue repair process. In terms of differentially expressed genes, the expression of some key genes in the WEV-bFGF group changed significantly: pro-inflammatory and pro-apoptotic substances such as *Il17rd*, *Casp2*, *Casp8*, *Casp7*, *Nfkbia*, *BCL212*, *Bak* and *Il6r* were down-regulated; On the contrary, genes encoding key components of the MAPK signaling pathway, such as *Mapk8*, *Mapk1*, *Mapk7* and *Mapk6* were upregulated. These changes in gene expression verified at the molecular level the mechanism by which WEV-bFGF synergistically promoted renal repair by regulating apoptosis, inflammation and cellular signal transduction networks. Compared with the use of bFGF alone, WEV-bFGF further markedly upregulated anabemy-related pathways such as PPAR, estrogen, stem cell pluripotency regulation and sphingolipid metabolism, while inhibiting immune-inflammatory signaling pathways such as TNF, IL-17, Toll-like receptors and chemokines, thereby more effectively improving the regenerative effect. In addition, the pathways of cellular senescence and apoptosis were also downregulated. These results indicated that WEV-bFGF could promote the regenerative microenvironment by enhancing anabolic processes, but also exert therapeutic potential through synergistic anti-inflammatory effects.

Finally, WEV-bFGF was shown to enable bFGF to target ischemic renal tissue rapidly and efficiently, but whether it could impact the KIM-1-related pathway was unknown. KIM-1 was involved in the recruitment and activation process of inflammatory cells, which might further exacerbate kidney damage [28, 29]. It was a key molecular node connecting injury recognition, inflammation regulation and tissue repair, providing a potential target for the clinical treatment of AKI [30]. Notably, the anti-KIM-1 antibody RMT1-10 accumulates specifically in proximal tubules where KIM-1 is highly expressed, thereby attenuating lupus nephritis through T-cell modulation [28, 31]. However, previous studies also reported the extracellular IgV domain of KIM-1 had the ability to recognize phosphatidylserine (PS) [32], which could mediate phagocytic clearance of apoptotic cells and thereby alleviate the local inflammatory microenvironment. In addition, KIM-1 could also activate pro-survival signaling pathways such as extracellular signal-regulated kinases ERK/MAPK [33] and PI3K/AKT [34], promoting the proliferation and migration of proximal tubular epithelial cells (PTECs). Therefore, the effect of WEV-bFGF on KIM-1 expression and related signaling pathways requires further investigation. In addition, long-term studies are necessary to evaluate the sustained therapeutic efficacy and safety profile of WEV-bFGF. These limitations should be addressed in future research.

## Conclusion

This study identified a novel KIM-1-targeted peptide-WEV peptide using computational design and optimization strategies, which had superior specificity and binding affinity with KIM-1 *in vitro* and *in vivo*. Then, a KIM-1-targeted recombinant WEV-bFGF protein was constructed to direct bFGF specifically to the ischemic kidney, where KIM-1 was highly expressed, thereby enhancing its accumulation and retention within the hypoxic microenvironment. This targeted delivery of WEV-bFGF could effectively protect ischemic kidney, decrease cell apoptosis and inflammatory response, alleviate fibrosis and improve renal function. This process was revealed to activate *Mapk1*, *Bcl212* genes, while inhibiting *Casps*, *Il6r* genes by transcriptome analysis. This study provided novel insight for the screening of targeted peptides and establishing a KIM-1-targeted bFGF delivery system for the repair of AKI.

## Supplementary Material

rbag050_Supplementary_Data

## Data Availability

Data will be made available on reasonable request.
