# Supplementary material for: AI-guided design and optimization of a novel KIM-1-targeted peptide for bFGF delivery in acute kidney injury repair
Source: Regen Biomater. 2026 Mar 9;13:rbag050. doi: 10.1093/rb/rbag050 (PMC13075985; doi:10.1093/rb/rbag050)
Supplement: rbag050_Supplementary_Data [file rbag050_supplementary_data.docx]

**
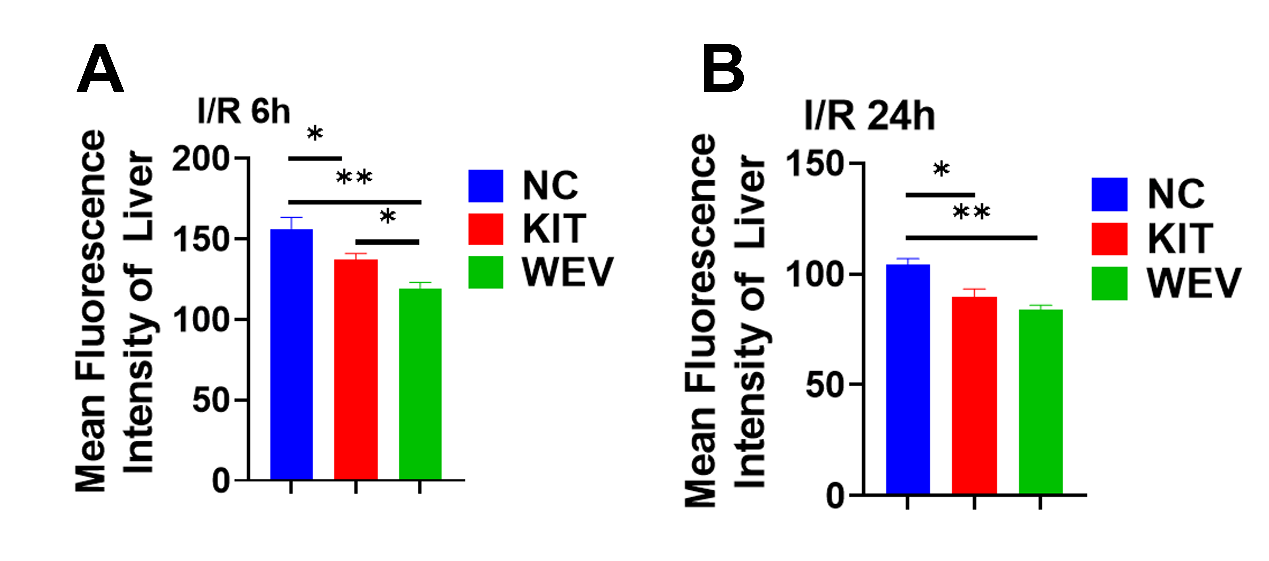
 Supplementary Figure 1:** (A) Statistics of the average fluorescence intensity in the liver 6 h after injection of the targeted peptide; (B) Statistics of the average fluorescence intensity in the liver 24 h after injection of the targeted peptide; All quantitative data are presented as mean ± SD, * *p* < 0.05, ** *p* < 0.01, *N* = 6.


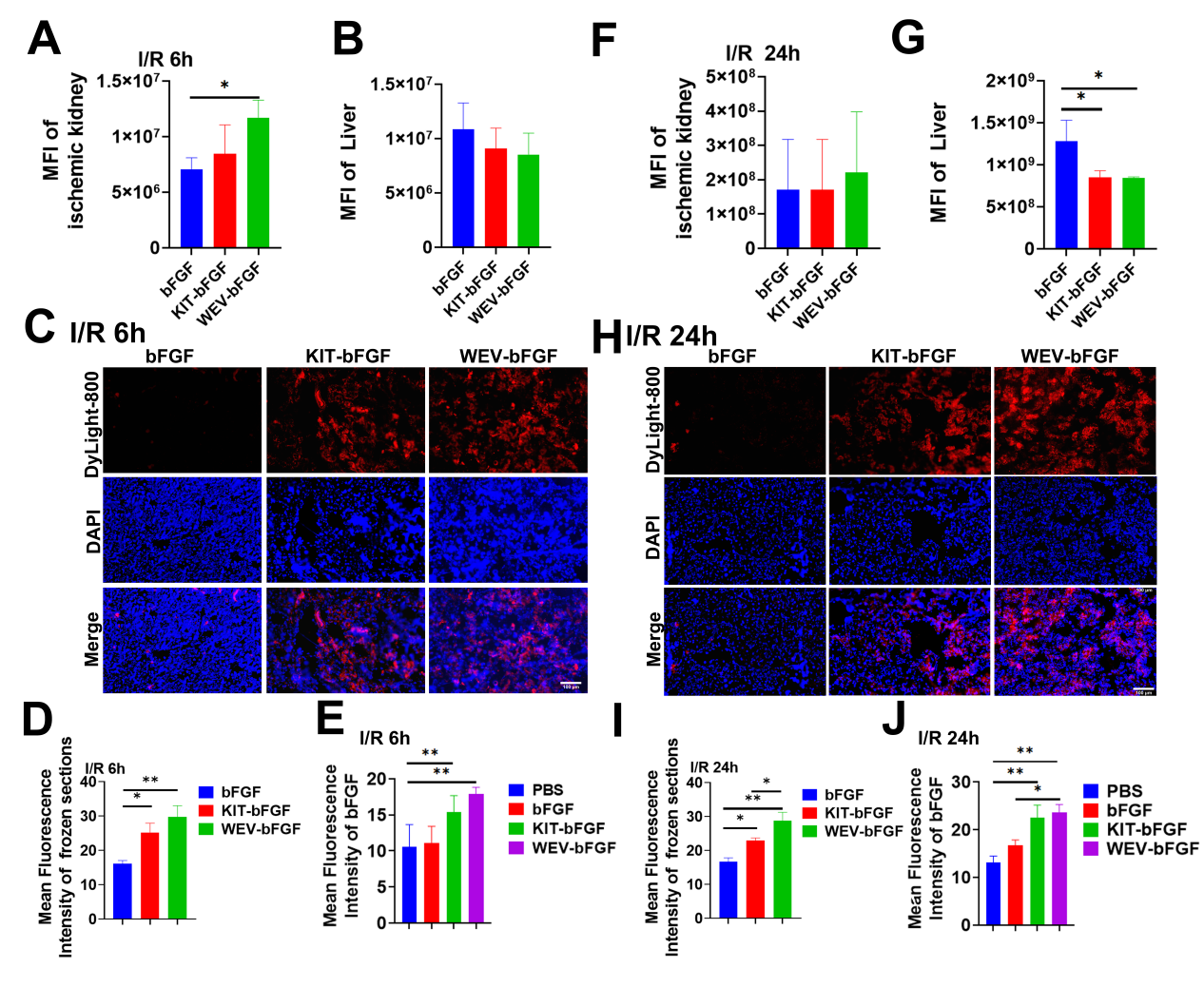


**Supplementary Figure 2:** (A) Statistical analysis of fluorescence intensity of Dylight800-labeled recombinant proteins in ischemic kidneys at 6 h after administration; (B) Fluorescence distribution in the liver of acute renal ischemic injury 6 hours after injection;(C) The results of frozen sections showed the distribution of targeted protein on the surface of ischemic renal tissue 6 h after I/R injury, with a scale of 100 μm; (D) Statistics of fluorescence intensity of frozen sections 6 h after administration; (E) Statistics of the Mean fluorescence intensity of bFGF in immunofluorescence staining 6 h after protein injection; (F)Statistical analysis of fluorescence intensity of Dylight800-labeled recombinant proteins in ischemic kidneys at 24 h after administration; (G) Fluorescence distribution in the liver of acute renal ischemic injury 24 h after injection; (H) The results of frozen sections showed the distribution of targeted protein on the surface of ischemic renal tissue 24 h after I/R injury, with a scale of 100 μm; (I) Statistics of fluorescence intensity of frozen sections 24 h after administration; (J) Statistics of the Mean fluorescence intensity of bFGF in immunofluorescence staining 24 h after protein injection; All quantitative data are presented as mean ± SD, * *p* < 0.05, ** *p* < 0.01. *N* = 6.


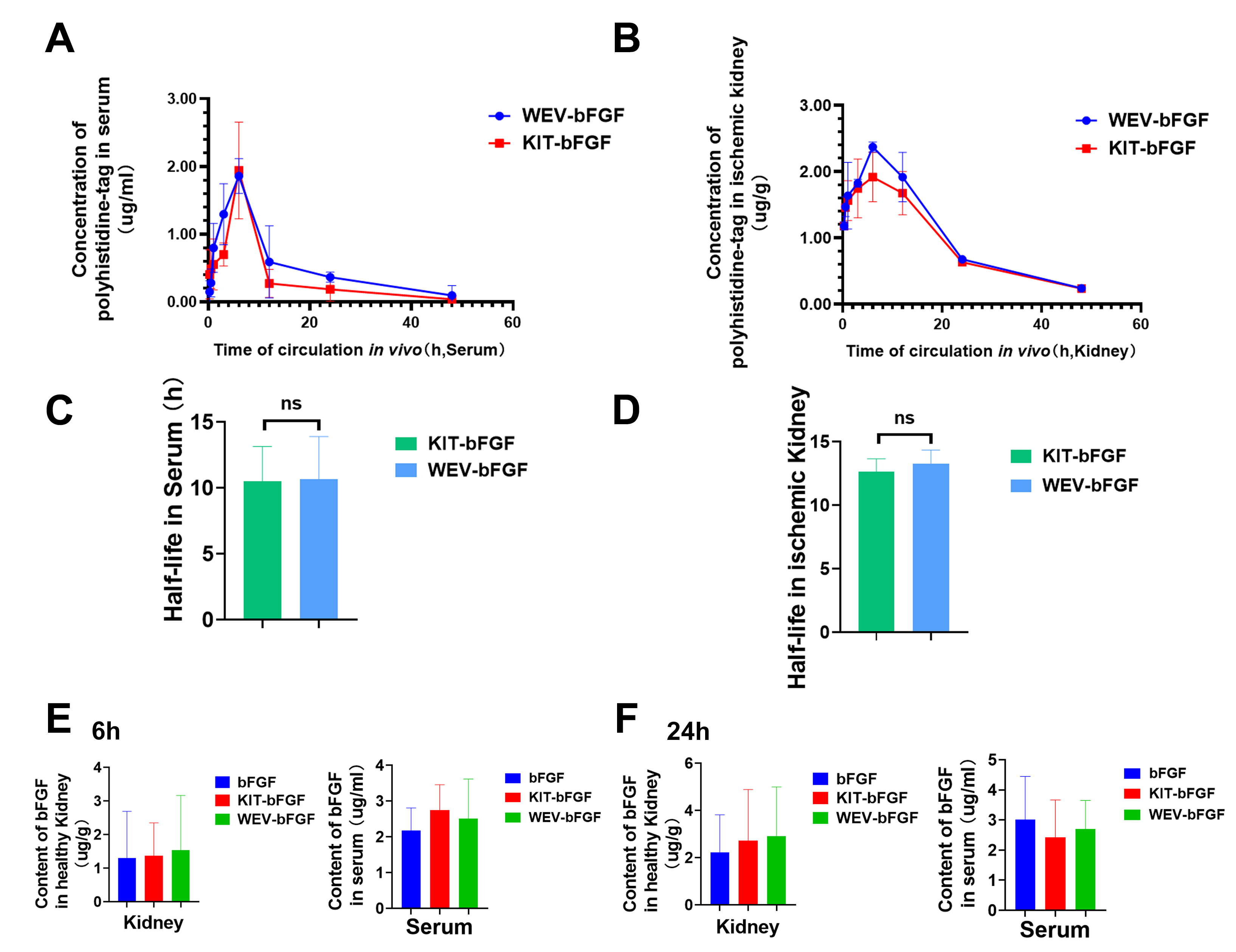
**Supplementary Figure 3:** (A) Serum concentration-time profiles of bFGF following intravenous injection of WEV-bFGF or KIT-bFGF in AKI model rats, as determined by ELISA. (B) Renal tissue concentration-time profiles of bFGF in ischemic kidneys of AKI model rats after intravenous injection of WEV-bFGF or KIT-bFGF. (C) Calculated serum half-life of bFGF derived from WEV-bFGF or KIT-bFGF. (D) Calculated renal tissue half-life of bFGF in ischemic kidneys following administration of WEV-bFGF or KIT-bFGF. (E) Serum and normal kidney concentrations of bFGF at 6 h after tail vein injection of WEV-bFGF or KIT-bFGF in healthy rats. (F) Serum and normal kidney concentrations of bFGF at 24 h after tail vein injection of WEV-bFGF or KIT-bFGF in healthy rats. *N* = 6

| **Gene name** | **Direction** | **Sequence** |
| --- | --- | --- |
| *GAPDH* | FORWARD | GGCAAGTTCAACGGCACAGTC |
|  | REVERSE | TCGCTCCTGGAAGATGGTGATG |
| *Mapk1* | FORWARD | GGCTGTTCCCAAATGCTGACTCC |
|  | REVERSE | TTGAATGGTGCTTCGGCGATGG |
| *Mapk6* | FORWARD | TGAGCCAGTAGAGGATGGGAAGC |
|  | REVERSE | GAAACTGTGGGATGCCTATGGACTC |
| *Bak* | FORWARD | GGACGACATCAACCGACGCTATG |
|  | REVERSE | AACAGGCTGGTGGCAATCTTGG |
| *Nfkbie* | FORWARD | GTCAAGGAACCACAGGAGAAGGAAG |
|  | REVERSE | GGTGTAGGTGAGCGAGGAGGAG |
| *Fgf2* | FORWARD | CATTAGAGCCAGAAGAGCCACCAAC |
|  | REVERSE | TCACGGCGGCATCTTTCAACAG |
| *Bcl212* | FORWARD | ATGGCGACCCCAGCCTCAA |
|  | REVERSE | TCACTTGCTAGCAAAAAAGGCC |
| *Casp8* | FORWARD | GCAAAGGAAGCAAGAACCCATCAAG |
|  | REVERSE | TGAGCCCTGCCTGGTGTCTG |
| *iL-6r* | FORWARD | ATGCTGGCCGTCGGCTGC |
|  | REVERSE | CTATCTGGGGAAGAAGTAGTCT |

**Supplementary Table 1:** The primer sequence was used for quantitative PCR (qPCR), and its amplification efficiency was verified.
